# Information-seeking behaviours and uncertainty around accessing primary care in the changing landscape of the COVID-19 pandemic: a qualitative study

**DOI:** 10.3399/BJGPO.2021.0151

**Published:** 2022-02-09

**Authors:** Lynsey Rachael Brown, Andrew James Williams, Kevin Shaw, Gozde Ozakinci, Mara Myrthe van Beusekom

**Affiliations:** 1 School of Medicine, University of St Andrews, St Andrews, UK; 2 Independent Scholar, San Francisco Bay Area, California, USA; 3 Faculty of Natural Sciences, University of Stirling, Stirling, UK

**Keywords:** health information, care seeking, SARS-CoV-2, COVID-19, primary health care

## Abstract

**Background:**

The COVID-19 pandemic has had and will continue to have a disproportionate effect on the most vulnerable. Public health messaging has been vital to mitigate the impact of the pandemic, but messages intended to slow the transmission of the virus may also cause harm. Understanding the areas where public health messaging could be improved may help reduce this harm.

**Aim:**

To explore and understand health communication issues faced by those most likely to be impacted by the COVID-19 pandemic.

**Design & setting:**

A qualitative study using online surveys. The area of focus was Fife, a local authority in Scotland, UK.

**Method:**

Two consecutive surveys were conducted. Survey 1 explored the observations of support workers and Facebook group moderators, and focused on key issues faced by service users, as well as examples of good practice (*n* = 19). Survey 2 was aimed at community members, and focused on issues regarding access to and communication around access to primary care (*n* = 34).

**Results:**

Survey 1 found broad issues around communication and access to primary care services. Survey 2 emphasised key issues in accessing primary care, including: (a) the lengthy process of making appointments; (b) feeling like a burden for wanting to be seen; (c) a lack of confidence in remote triaging and consultations; and (d) not knowing what to expect before getting an appointment.

**Conclusion:**

Clear issues regarding access to primary care were identified. The new understanding of these issues will inform a co-creation process designed to develop clear, actionable, and effective public health messages centred on improving access to primary care.

## How this fits in

The pandemic has had a disproportionate impact on vulnerable communities, and there must be an understanding of what issues contribute to this impact. This study improves this understanding and identifies key issues experienced by individuals that negatively impact their ability to access primary care, likely leading to future health problems. This study is relevant and of interest to clinicians, as it provides insight into the issues experienced by patients, which can in turn be fed into practice.

## Introduction

The COVID-19 pandemic has had a profound impact on the mental, physical, and social wellbeing of individuals.^1–3^ Until the roll out of the vaccine programme, limiting the spread of the virus was the most critical intervention available to reduce mortality and transmission of COVID-19. To achieve this, several public health measures and regulations were put in place, including compulsory face coverings, social distancing, shielding, and self-isolation strategies. Messages supporting these regulations were implemented, including the *FACTS* campaign^4^ in Scotland (Face coverings, Avoid crowds, Clean hands, Two-metre distance, and Self-isolate) and similar *Hands, Face, Space* campaign in England.^5^ Limiting the spread of the virus has been vital, yet some of the regulations and messages implemented to achieve this — for example, self-isolation and shielding — also adversely impact the lives of the general population and, more acutely, the most vulnerable in society.

The pandemic has produced many challenges around communication in terms of reliability of the messages, trust in scientific research, reach of key messages, and effects on behavioural outcomes.^6–8^ There has been some discontent and confusion from the public regarding some of these key campaigns, such as struggling to remember what the *FACTS* campaign acronym stood for.^9^ Such difficulty and confusion renders the messages unactionable. There is also evidence of confusion owing to incomplete, ambiguous, and/or inconsistent messaging from the government, including via mainstream media.^10–13^ Such confusion has been associated with increased mental health problems, including increased depression and anxiety.^14^

Moreover, rapidly changing recommendations and feelings that imposed regulations are 'unreasonable' or 'unnecessary' have led to advised behaviours not always being adhered to.^15^ Michie *et al* ^16^ suggest people are left feeling unsure about what to do when messages are not ‘actionable’ enough. There have also been many sources of misinformation, as currently seen in the context of vaccination safety, a phenomen which has been labelled the ‘infodemic’.^17–19^ These communication issues have had and will continue to lead to a variety of negative consequences, which will impact society for years to come. These consequences include increased risk from COVID-19, increased levels of anxiety, and delayed health-seeking behaviours, which will likely lead to an influx of serious health conditions further down the line and unnecessary mortality.^3,20^

Despite advances with the vaccination programme, it is likely that health messaging about transmission risk-reducing behaviours will continue to play a key role in managing the pandemic,^8,16^ ameliorating the issues associated with the ‘infodemic’, and improving adherence to preventive measures.^12^ It is therefore necessary to ensure the messages are actionable and clear, particularly for those disproportionately affected by the pandemic. In Scotland, deprivation is a key contributing factor to health inequalities and increased COVID-19 impact, with those living in more deprived areas more likely to be admitted to hospital with COVID-19.^21^ Other groups are also disproportionately impacted in the UK, including older adults, people with lower income, and those from South Asian and Black communities.^22,23^ Such disproportionate impact highlights and exacerbates current health inequalities,^24^ which must be tackled.

Throughout the pandemic, the importance of communities has been emphasised and, in particular, the necessity for services and agencies to collaborate with communities to enable knowledge exchange, and the development of services and solutions.^25^ People have self-organised into community groups to support each other. This can involve finding relevant information and even collaborating to develop messaging by, for example, collating and providing information on available services in the local area.^26^ Local organisations, support workers, and community Facebook groups have been key contacts assisting individuals and helping with the spread of information.^27^ In addition, public health authorities globally have increasingly used Facebook for dissemination of messages and information during the COVID-19 pandemic, with varying levels of success.^28^

In the constantly changing ‘new normal’, there are also constantly changing communication needs. There is an opportunity to purposefully make use of the insights that such online groups have into the key information needs of their own communities. Intentionally incorporating local experience can also encourage the development of messages that are relatable, relevant, and actionable, which is essential to support behaviour change.^29,30^ In addition, partnerships between community leads and public health authorities help to ensure that messages reach underserved communities.^31^ This is important as people who are disadvantaged by health inequalities and have less access to information are less likely to comply with public health recommendations.^32^

The aim of this work was to gain insight into relevant and priority issues with health communication, to help determine the focus for a community-embedded co-creation process that develops clear and relevant messages to support vulnerable groups.

## Method

Two consecutive online surveys were distributed among community groups and services. These surveys were undertaken with the aim that they would serve as the basis of a broader co-creation process.

Survey 1 aimed to gain insight into what information needs support workers observed among the communities they serve. The findings of this survey informed the aim of survey 2, which was to get a fuller picture of communication issues around access to primary care from a diverse set of community members potentially at increased risk of negative outcomes from the pandemic.

The components of the Consolidated criteria for reporting qualitative research (COREQ) checklist^33^ relevant to qualitative research using surveys (that is, Domain 2: Study design and Domain 3: Data and analysis) were consulted to ensure best practice when describing the study.

### Surveys

#### Survey 1: Support workers

Survey 1 consisted of seven open-ended questions exploring the observations of support workers on the following: information-seeking behaviours in their communities; helpful communication and examples; and unmet needs of community members. Participants were also asked for their views about channels other than Facebook where people found health information and support. The support workers' views were expected to be a reliable representation of conversations in the community, as they were in contact with community members throughout the pandemic and had a unique opportunity to gain insight into issues experienced both online and offline. This survey was developed and piloted with the wider project team, including representatives from Public Health and Health promotion; a third sector interface; local council; and a community representative. Feedback from the community representative played a key role in shaping the questions to effectively capture possible issues. The outcomes of survey 1 were also validated in survey 2, ensuring reliability.

#### Survey 2: Community members

Survey 2 consisted of 16 questions. These focused on communication around accessing primary care, including making appointments with the GP practice, attending remote and/or in-person consultations, and picking up prescriptions. Questions alternated between closed responses on a 5-point Likert scale (ranging from ‘not at all’ to ‘extremely’ or when appropriate from ‘very difficult’ to ‘very easy’) and open-ended questions exploring responses to the closed questions. Data were also collected on the service or Facebook group used to access the survey, as well as the respondent’s age, sex, ethnic group, history of chronic illness, shielding status, and COVID-19 vaccination status. Demographic measures were self-reported and open to participants' interpretations, which is recommended practice to ensure the participant did not feel pressured to label themselves in a way that felt uncomfortable to them. This survey was developed collaboratively like survey 1, with additional involvement of a user-experience researcher (KS), with the aim to extrapolate and validate the identified issues around primary care.

### Recruitment

#### Research location: Fife

Fife is a county in Scotland with a population of approximately 370 000.^34^ Its demographic characteristics are broadly similar to that of Scotland as a whole, but the percentages of older adults and younger people are greater. The level of deprivation is also slightly higher than that of Scotland as a whole, increasing the population’s risk from the impact of COVID-19.^35^ The majority of the population of Fife (over 90%) are described as White, indicating limited ethnic diversity in the region.^36^

#### Survey 1: Support workers

Survey 1 was sent via email to a small number of community learning and development, social work, and health contacts linked to the planning group, as well as to moderators of Facebook community and support groups, both through existing contacts with NHS Fife and Fife Health Promotion service. This pragmatic sampling method did not allow for calculation of a response rate but was chosen to increase the likelihood of recruiting participants at a time when support workers were under a great deal of pressure.

Seventeen support workers from community services, housing, social work, and voluntary organisations, and two moderators for local Facebook groups responded (June–September 2020).

#### Survey 2: Community members

To recruit to survey 2, a distribution list maintained by Fife Health Promotion service of Facebook moderators of community groups and service leads was used. Individuals were asked to share the survey with their groups. This survey was completed by 34 community members from 10 Facebook groups and local community services (March–April 2021). To improve the likelihood of a diverse representation, participants were also recruited through equalities and faith groups. The resulting set of participants represented a diversity of people with respect to age, Scottish Index of Multiple Deprivation (SIMD: deprivation scores for each area of Scotland based on a variety of factors),^37^ and history of chronic illness. Four men and 30 women responded. Nine of the 34 participants had been shielding during the pandemic, and 24 had received their COVID-19 vaccination. The majority described their ethnic group as White (70.6%). The remainder described their nationality instead of ethnic group, being Scottish or British (23.5%), European (2.9%), Scottish Chinese (2.9%). For a full description of demographics, see Table 1.

**Table 1.** Description of demographic characteristics of the sample (*n* = 34).

Variable	*n* (%)
Age, years	
Mean (SD)	51 (11.3)
Range	20–67
Sex	
Male	4 (11.8)
Female	30 (88.2)
SIMD	
Quintile 1–2	10 (29.4)
Quintile 3	8 (23.5)
Quintile 4–5	10 (29.4)
Missing	6 (17.6)
History of chronic illness	
Yes	17 (50.0)
No	17 (50.0)
Shielding	
Yes	9 (26.5)
No	25 (73.5)
COVID-19 vaccination	
Yes	24 (70.6)
No	10 (29.4)
Ethnic group as described by participants	
White	24 (70.6)
British or Scottish	8 (23.5)
Scottish Chinese	1 (2.9)
European	1 (2.9)

SIMD = Scottish Index of Multiple Deprivation

### Analysis

#### Survey 1

Data collected were extracted to Excel (version 2108) and analysed thematically,^38^ with each key issue identified representing a theme. A summary of each key issue was created with appropriate evidence from the survey attributed to these. Issues were often explicitly stated by the support workers and so minimal interpretation was required. The outcomes of this survey were discussed and validated with the project group to prioritise the scope of the second survey.

#### Survey 2

Data collected were extracted to Excel (version 2108). Qualitative data were analysed thematically.^38^ This process involved identifying key themes, representing issues and/or examples of good practice experienced when accessing primary care. To identify and develop these themes, data were first read and re-read by MvB and LB. The data sheet was annotated, denoting potential issues experienced and examples of good practice, drawing on both deductive (results from the previous survey) and inductive means (new knowledge from the survey as interpreted by the researchers). These points were then discussed to condense where possible. Finally, a coding table was created denoting each of these themes and associated evidence. Descriptive statistics and frequencies were conducted to enable description of the quantitative data collected.

## Results

### Survey 1: Support workers

The surveyed support workers served a wide range of individuals including the following: older adults on low income; young people; lone parents; vulnerable families; young carers; people in adult basic education; and groups affected by drug and alcohol use, homelessness, trauma, mental health issues, and unemployment. Information-seeking behaviours and information needs within these groups were captured in three themes, in order of descending frequency: (1) accessing health services; (2) COVID-19 transmission and protection; and (3) mental health support. As accessing health services was the most dominant theme and issue, it was agreed this would be the focus of survey 2 (community members).

#### Accessing health services

Information was sought on what services were open, and in particular access to primary care, appointments, waiting times, referrals, and medication review. In addition to *'confusion around* [the] *new way of working in primary care'* (support worker), other services that were mentioned in this context were mental health services, harm reduction around drug and alcohol use, pharmacies, and crisis support.

#### COVID-19 transmission and protection

Another common need in the groups was for information on COVID-19 transmission and on mitigating risks. Specific topics were: information on social distancing, advice on masks and personal protective equipment (PPE), COVID-19 testing, safety of children, and safety as part of the shielding group or as a carer.

#### Mental health support

Several responses specifically mentioned mental health support, including self-care such as diet, exercise, sleep, and coping with stress. In this context, tackling loneliness and sharing details of contact groups for support were also mentioned.

#### Message delivery

Support workers noted examples of good practice regarding messages on domestic abuse, harm reduction around alcohol and drug use, and support services in mental, sexual, and physical health. Community-driven spreadsheets of available services and contacts, listing available services, and overviews of essential contacts were mentioned as helpful formats. A respondent pointed out a need for NHS messages to be publicised and shared, while another said there was already too much information. A need for consistent, simple messages across platforms was described as desirable, as well as continued promotion of the core public health messages (for example, *FACTS* guidance) and clarity on differences around the UK and Scottish Government advice.

In terms of the accessibility of the available health messages, guidance from governing and organisational bodies was perceived as helpful, but sometimes too long. Easy-to-read information, roadmaps and simple messages were suggested instead. Posters, social media, TV and/or radio, and signposting via workers over the phone were mentioned as channels that worked well to help spread and reinforce messages. Opportunities were identified to address digital barriers and at the same time increase engagement on Facebook through infographics and videos.

### Survey 2: Community members

Results indicated that while 41.2% of the respondents were not at all hesitant to make a GP appointment, 35.3% were ‘very–extremely’ hesitant. A key factor appeared to be the difficulty of actually getting an appointment; for example, almost one-third did not find this process clear at all. Two respondents commented on the use of online forms to obtain an appointment: one felt uncomfortable explaining the reason for their appointment; and the other was dissatisfied with having to wait for a response after completing the online form. However, most issues related to making appointments via the telephone. The main concern for people was the total length of the process: in the first instance to get through from the answering system to the receptionist, then to triaging, and finally to be allocated an appointment:

*'… waiting to get through can be frustrating. I recently took 48 attempts to get through.* […] *if you don't keep trying there are no appointments left.'* (Female, 50-years-old)

However, others found themselves unable to get through or dismissed:

*'Had the phone hung up on me as my appointment request was not urgent.'* (Male, 20-years-old)

These respondents expressed their frustration with this process and trying to make an appointment was described as pointless. Another barrier that various people experienced was feeling ‘unwelcome’, or *'your* [sic] *made to feel like a burden … for expecting to see your GP'* (Female, 40-years-old). Confusion around when it is appropriate to contact the GP was apparent: *'Adverts say see your GP. Local info and that from surgery say no'* (Female, 54-years-old). As a result of this message, some respondents described putting off making appointments to avoid burdening resources. A third reason that was provided for not making an appointment was the (perceived) difficulty of being referred for follow-up care.

One respondent also mentioned mental health concerns as a reason for feeling hesitant. However, another mentioned the manner in which their mental health concerns had been dealt with as the reason they did not feel hesitant at all:

*'Whole process of keeping in contact* [about mental health] *and changing medication went very smoothly.'* (Female, 50-years-old)

Finally, a respondent mentioned being *'* *scared* *…* [to] *catch COVID* *'*, as well as worried about the outcome of an appointment, for instance, *'* *finds something like* *…* *cancer* *'* (Male, 60-year-old), emphasising fear as a barrier to health-seeking behaviours. Although there were concerns about catching COVID-19, others mentioned being satisfied with the COVID-19 safety measures in place, leading to reduced hesitancy around making and attending appointments.

More than half of the respondents considered attending the consultation easy *'once you get an appointment'* (Female, 54-years-old). Most (29.4%) had received a phone consultation, while a further 29.4% had received both a phone and face-to-face consultation, 11.8% had received face-to-face only and none had received a video consultation. A recurring issue mentioned was not getting a fixed time for the appointment but rather a time range. Some indicated experiencing stress owing to not being able to see their regular GP, as they struggled to talk with other healthcare professionals. There was also dissatisfaction around not having a choice in the type of appointment. While some described phone consultations as easy and comfortable, others felt a lack of confidence in remote appointments and triaging:

*'My problem is something that needs to be seen not described. I was asked to send a photo but it’s not showing on the photo. I just want to see someone and I can't. I am having to wait over 2 weeks just to speak to someone and this is not acceptable to me when I'm in pain.'* (Female, 51-years-old)

This confidence was also questioned in light of prescriptions by two respondents, with one saying that *'I seem to have had a prescription given by a receptionist and pharmacist but haven't spoken to a doctor*' (Female, 55-years-old). However, overall the process of picking up prescriptions was said to be very to extremely clear (70.6%), with the integrated process between GP and pharmacy being mostly unchanged.

While just 11.8% experienced their appointment as ‘very difficult to attend’, 26.5% felt it was not at all clear what they could expect from attending an appointment. Most people found information on how to access their GP practice on the practice website (35.3%) or over the phone (20.6%), while a further 8.8% used social media, 2.9% got information from their family and/or friends, 2.9% from a leaflet, and 2.9% from a maternity app. In addition, 23.5% of respondents used a combination of these media to find information about their practice. It was suggested that information on the consultation could have been emailed or sent in between making the appointment and receiving the consultation.

## Discussion

### Summary

This study aimed to gain insight into information needs present in the community, as observed by support workers and local Facebook support groups. The results show that access to primary care continues to be a relevant and urgent problem, even this far into the ‘new normal’. The main barriers for people to contact their GP appear to be the lengthy process of making appointments, feeling like a burden for wanting to be seen, a lack of confidence in remote triaging and consultations, and not knowing what to expect before getting an appointment.

### Strengths and limitations

Using online surveys — a practical result of the pandemic — likely excluded those who experience barriers to accessing technology. The effect of this was mitigated by surveying support workers who work with these groups. Moreover, it was not possible to ascertain the number of individuals who were sent and/or interacted with the survey. Regardless, this was the best suited recruitment method owing to the broad net cast at a time when recruitment was generally difficult. This enabled the involvement of vulnerable populations and follow-up work in a timely manner. This study looked at local information needs in a relatively small group; thus, the sample is not necessarily widely generalisable. However, with the demographic characteristics of Fife being broadly similar to that of Scotland, some careful extrapolations can be made.

Despite attempts to recruit a more ethnically diverse population, the majority of participants described themselves as White. The population recruited, however, is similar to that of the area of Fife more generally, with over 90% of the population being White.^36^ Also, a number of participants reported their nationality as opposed to ethnic group, leading to a number of missing data points. However, self-report demographics were recommended to ensure participants felt comfortable responding, which is key in collaborative work. Moreover, this study was successful in reaching those living in areas of high deprivation, which is a contributing factor for negative impact from COVID-19 in Scotland.^21^

### Comparison with existing literature

A significant increase in serious health problems is anticipated as a result of people’s changed health-seeking behaviours since the start of the pandemic.^20^ Recent campaigns have attempted to target these problems; for example, the campaign to create awareness of lung cancer symptoms.^39^ However, public messages such as *'General practices are very busy, so before deciding to contact your practice, please think whether you can manage your problem yourself''* ^40^ may feed into people’s concerns about ‘burdening’ their GP, rather than empowering people with potentially serious health problems to seek out support.

This fear of contracting COVID-19 when accessing primary care services, in particular, has been highlighted in mainstream media, as well as previous research,^41,42^ and this study. Therefore, it is necessary to ensure future messages support access to primary care, alleviating this sense of burden and worry, and in turn reducing the anticipated '*tsunami of demand* *'*.^43^ A recent campaign by NHS England, *Help Us, Help you*, aimed to encourage individuals to access healthcare services, with the first phase focusing on contacting their GP.^44^ However, the key phrasing of *Help Us, Help You* may continue to put some people off, as it places the onus and burden of responsibility on the patient without much specific guidance.

Various studies have looked at the use of telehealth and consequences on patient uptake of appointments. In the past, telehealth has often been adopted by remote communities; the pandemic has led to a widespread adoption of telehealth systems.^45^ It is expected that even after social distancing regulations are relaxed, this new way of working will play an important role in general practice.^46^ However, this study shows that there is still work to be done to help manage patients’ expectations around such consultations and ensure they are confident in accessing services.

In addition to the increase in telephone consultations, practices have seen a significant influx in calls to schedule appointments. The issues observed in this study around the process of making appointments are likely to vary from practice to practice. Regardless, the study does emphasise the important role of GP practice receptionists as the key contact point for patients, and the potential need for additional resources and support to help them manage patients’ expectations of the consultation process. Over the years, various studies have highlighted the often overlooked but essential role of these frontline administrative staff.^47–49^

The results of the present study corroborate the findings of a recent HealthWatch report highlighting the benefits of telehealth for some and the difficulties it poses for others.^46^ The difficulties emphasised revolved around the consultation itself, but also included access to primary care, indicating that many feel services are not *'* *open for business* *'*.^46^ This also highlighted how patients can feel like a burden on the NHS so choose to avoid making appointments, leading to visits to accident and emergency (A&E) and advanced illness.^46^ It is clear from this study’s results that these issues are still prevalent, and work is required to develop means to educate and inform individuals, through messaging, about access to primary care services. Improved communication in this area could reduce the future burden on the NHS and reduce the anticipated increase in health inequalities.

### Implications for practice

Access to primary care is a continuing issue and potentially vulnerable groups could benefit from supportive communication for the foreseeable future. Issues include: (a) the lengthy process of making appointments; (b) feeling like a burden for wanting to be seen; (c) a lack of confidence in remote triaging and consultations; and (d) not knowing what to expect before getting an appointment. The findings from this study will be used to inform an online, community-embedded co-creation process to create messages to support access to primary care for the community and provide communication guidance for practices.
